# Deciphering the molecular clock: exploring molecular mechanisms and genetic influences on skin ageing

**DOI:** 10.1007/s10522-025-10296-x

**Published:** 2025-08-02

**Authors:** Horng Yih Ng, Yuan Seng Wu, Mohitosh Biswas, Maw Shin Sim

**Affiliations:** 1 Department of Pharmaceutical Life Sciences, Faculty of Pharmacy, Universiti Malaya, 50603 Kuala Lumpur, Malaysia; 2 Precision Medicine & Omics Centre (PrOmiC), Faculty of Pharmacy, Universiti Malaya, 50603 Kuala Lumpur, Malaysia; 3 Department of Biomedical Sciences, Sir Jeffrey Cheah Sunway Medical School, Faculty of Medical and Life Sciences, Sunway University, Sunway City, Malaysia; 4 Sunway Microbiome Centre, Faculty of Medical and Life Sciences, Sunway University, Sunway City, Malaysia; 5 Department of Pharmacy, Faculty of Science, University of Rajshahi, Rajshahi, Bangladesh

**Keywords:** Skin ageing, Genetic association studies, Wrinkles, Skin sagging, Hyperpigmentation, Collagen metabolism

## Abstract

Skin ageing is a multifaceted process influenced by both intrinsic genetic factors and extrinsic environmental exposures. This review explores the genetic variants and molecular mechanisms underlying skin ageing phenotypes, while identifying gaps in current research to inform future studies. A systematic search of the Scopus and Web of Science databases was conducted, with articles screened based on criteria including a focus on human genetic association studies and indexing in either Scopus or the Institute for Scientific Information (ISI) Web of Science. The quality of included studies was assessed using the Q-Genie tool. Thirteen studies met the inclusion criteria, collectively analysing 115 single nucleotide polymorphisms (SNPs) across 99 genes. The *OCA2* gene emerged as the most frequently investigated, consistently linked to hyperpigmentation. Overall, findings indicate that skin ageing phenotypes are associated with genes involved in collagen metabolism, melanogenesis, oxidative stress, and mechanical properties of the skin. Notably, *SPATA33* rs35063026 and *IRF4* rs12203592 polymorphisms exhibit pleiotropic effects, contributing to both wrinkling and pigmentation changes. Interestingly, some variants, such as *SPTLC1* rs7042102 and *REEP3* rs11961184, display paradoxical effects, underscoring the complexity of genetic modulation. Genes implicated in extracellular matrix (ECM) degradation, such as *SMYD3*, and those responsive to environmental pollutants, like *CYP1A1*, were associated with increased skin sagging. Conversely, variants in genes such as *COL1A2* and *COL13A1*, which support ECM integrity and skin resilience, were linked to protective effects. Despite these insights, many genetic associations remain poorly understood, highlighting substantial gaps in knowledge and the need for more comprehensive genetic research into skin ageing.

**Supplementary Information:**

The online version contains supplementary material available at 10.1007/s10522-025-10296-x.

## Introduction

Skin ageing is a complex and multifactorial process influenced by intrinsic and extrinsic factors. Intrinsic ageing is characterised by a gradual decline in the proliferative ability of cells such as keratinocytes, fibroblasts, etc. (Zhang and Duan 2018). Conversely, extrinsic ageing is predominantly induced by environmental factors, with chronic exposure to ultraviolet (UV) radiation representing the major environmental factor (Langton et al. 2010). Key visible features of ageing skin include wrinkles, sagging, and hyperpigmentation (Shin et al. 2019), which not only affect aesthetics but also reflect underlying molecular alterations.

Wrinkles are folds or creases in the skin, typically forming as a result of the natural ageing process. They result from reduced dermal collagen levels and the formation of abnormal elastic fibres (Kim et al. 2016). They can be categorised based on their regions, such as crow’s feet and nasolabial folds. Crow's feet, also known as lateral canthal lines, appear at the outer corners of the eyes, while nasolabial folds are the creases that extend from the sides of the nose down to the corners of the mouth (Jeong et al. 2020).

On the other hand, skin sagging arises from a reduction in skin elasticity and a decline in collagen fibre mass, primarily due to decreased collagen I synthesis in the aged dermis (Imokawa and Ishida 2015). Eyelid sagging or dermatochalasis commonly affects middle-aged or elderly individuals, presenting both aesthetic concerns and functional issues such as visual impairment and ocular irritation (Laville et al. 2019). Histological analysis of sagging eyelid skin reveals a loss of elastic fibres and a disrupted collagen network (Nagi et al. 2011).

The progressive decline in melanocyte activity with age represents a key feature of cutaneous ageing, with studies demonstrating a 10–20% reduction in functional melanocytes per decade (Hakozaki et al. 2014). This depletion disrupts uniform pigment distribution, contributing to uneven skin tone. Paradoxically, chronic UV exposure helps preserve melanocyte numbers in photoexposed areas by sustaining inflammatory signalling, primarily driven by reactive oxygen species (ROS) and pro-inflammatory cytokines such as interleukin-1 alpha (IL-1α) and tumour necrosis factor-alpha (TNF-α) (Pillaiyar et al., 2017). The resulting hyperactivation of residual melanocytes, combined with impaired melanosome transfer to keratinocytes, manifests clinically as mottled dyspigmentation. Solar lentigines (SL), or age spots, are a hallmark of this process, characterised by localised epidermal hyperplasia and melanin accumulation due to upregulated melanogenic enzymes (e.g., tyrosinase) and aberrant Wnt/β-catenin signalling (Kang et al. 2021). These changes are further exacerbated by age-related declines in DNA repair capacity and antioxidant defences, creating a self-perpetuating cycle of oxidative stress and pigmentary irregularities.

Advancements in genomics, particularly genome-wide association studies (GWAS) and candidate gene studies, have identified genetic variants associated with skin ageing phenotypes. Genes such as Interferon Regulatory Factor 4 (*IRF4)*, Melanocortin 1 Receptor (*MC1R)*, Agouti Signalling Protein (*ASIP)*, and Basonuclin-2 (*BNC2)* are implicated in facial pigmented spots during ageing (Jacobs et al. 2015). Despite these findings, numerous genetic association studies have been conducted in recent years, revealing novel genetic links to skin ageing phenotypes. While some of these associations have biological interpretations, others remain unexplained. Hence, this review aims to systematically evaluate and synthesise the current evidence on genetic variants associated with skin ageing phenotypes and to explore the underlying biological mechanisms that may explain these associations.

## Methods

### Literature search and eligibility criteria

This review included only research articles indexed in Scopus or the Institute for Scientific Information (ISI) Web of Science to ensure the inclusion of high-quality, peer-reviewed literature. To capture the most recent advancements, only studies published between January 2014 and August 2024 were considered. In line with the review’s objectives, only human studies focused on genetic association research were included.

Conversely, conference and proceeding papers, commentaries, and letters were excluded to mitigate the risk of bias arising from incomplete reporting. Similarly, review articles were omitted to prevent duplication of data already extracted from primary studies cited within those reviews. Furthermore, animal studies and articles published in languages other than English were excluded to maintain the relevancy and consistency of the review.

## Search strategy

A systematic literature search was conducted using two electronic databases: Scopus and Web of Science. These databases were selected based on the inclusion criterion requiring that only articles indexed in ISI Web of Science or Scopus be considered. Search terms and keywords were developed from preliminary searches and combined using Boolean operators (e.g., AND, OR) to ensure a comprehensive review. Filters were applied to exclude non-English publications, studies published outside the 2014–2024 range, and non-research articles such as conference papers, proceedings, commentaries, letters, animal studies, and reviews.

The systematic review was conducted adhering to the Preferred Reporting Items for Systematic Reviews and Meta-Analysis (PRISMA) 2020 statement (Page et al. 2021). The search strategy used was as follows: ("skin ag*ng"OR"ag*ng skin"OR"photoaged") AND (“genetic association studies” OR “genome-wide association study” OR “GWAS” OR"candidate gene study"OR"genotype"OR"Single Nucleotide Polymorphism"OR"SNP"OR"haplotype"OR"heritability") AND (“genetic polymorphism” OR “allele” OR “gene* varia*” OR “mutation” OR “genes” OR “chromosome” OR “genetic predisposition” OR “genetic susceptibility”).

Variations in electronic databases resulted in distinct field labels, specific operators, and syntax requirements. For example, the search query"All Topics"in Web of Science is equivalent to the"TITLE-ABS-KEY"parameter used in Scopus, as both refer to title, abstract, and keyword searches of the articles. Consequently, tailored search queries across different electronic databases are necessary to optimise information retrieval. The queries used in Web of Science and Scopus are described in Table 1. The online search was broadened by evaluating the bibliographic references of the eligible full-text articles.

**Table 1:** Search Query for Web of Science and Scopus

Electronic Database	Query
Web of Science	"skin ag*ng"OR"ag*ng skin"OR"photoaged"(Topic) and “genetic association studies” OR “genome-wide association study” OR “GWAS” OR"candidate gene study"OR"genotype"OR"Single Nucleotide Polymorphism"OR"SNP"OR"haplotype"OR"heritability"(All Fields) and “genetic polymorphism” OR “allele” OR “gene* varia*” OR “mutation” OR “genes” OR “chromosome” OR “genetic predisposition” OR “genetic susceptibility” (All Fields) and Article (Document Types) and Dermatology (Web of Science Categories)
Scopus	(TITLE-ABS-KEY ("skin ag*ng"OR"ag*ng skin"OR"photoaged") AND ALL ("genetic association studies"OR"genome-wide association study"OR"GWAS"OR"candidate gene study"OR"genotype"OR"Single Nucleotide Polymorphism"OR"SNP"OR"haplotype"OR"heritability") AND ALL ("genetic polymorphism"OR"allele"OR"gene* varia*"OR"mutation"OR"genes"OR"chromosome"OR"genetic predisposition"OR"genetic susceptibility")) AND PUBYEAR > 2013 AND PUBYEAR < 2025 AND (LIMIT-TO (EXACTKEYWORD,"Human") OR LIMIT-TO (EXACTKEYWORD,"Humans"))

## Study selection and data extraction

The screening process began with collating all identified records from the selected databases into Rayyan (Ouzzani et al. 2016), which is an online research collaboration platform designed to help researchers working on systematic reviews and other knowledge synthesis projects. After removing duplicates, the screening process was conducted by two reviewers. Initial screening was based on article titles and abstracts to assess relevance to the scope of this review. All potentially eligible studies then underwent full-text evaluation to determine inclusion. A systematic review of the reference lists (bibliographies) of included articles was also performed until no additional relevant studies were identified. Inter-rater agreement was established throughout the screening process to ensure consistency and reliability.

The extraction phase was established to gather relevant data from each study systematically. The following data were extracted from each eligible article: year of publication, author, sample size and subject demographics, country, gene, polymorphism, skin ageing phenotype and result. The first reviewer performed the data extraction, which was independently verified by the second reviewer.

## Quality assessment

The Quality of Genetic Association Studies (Q-Genie) tool was employed to evaluate the quality and risk of bias in genetic association studies (Sohani et al. 2015)This process ensures the methodological rigour and reliability of studies included in the systematic review. The tool consists of 11 items, and each item was rated using a 7-point Likert Scale (1 = poor, 3 = good, 5 = very good, 7 = excellent). Each item evaluates key aspects of genetic association studies, as mentioned in Table 2.

**Table 2:** Q-Genie Tool Items and Description

No	Assessment Item	Description
1	Rationale for Study	Determine whether a clear scientific rationale is provided for the selection of genes or justification for hypothesis-free GWAS
2	Outcome Definition	Assess whether the outcome (e.g., skin ageing traits) is appropriately defined, participants are appropriately sampled, and outcome assessors are blinded to the genotype status
3	Comparison Groups	Evaluate whether the control group are appropriately defined, controls are appropriately sampled to minimise selection bias, a detailed description of the selection procedure is outlined, and outcome assessors are blinded to the genotype status
4	Technical classification of the exposure	Determine whether the DNA source and storage methods are appropriate, genotyping techniques are valid, error rates and call rates meet genotyping quality thresholds, and Hardy–Weinberg equilibrium is tested and reported in control groups
5	Non-technical classification of the exposure	Determine whether genotyping was performed consistently across all samples, assessors were blinded while conducting genotyping, and samples were randomised to prevent batch-related biases
6	Other sources of bias	Assess whether the study acknowledges and discusses additional biases besides of selection bias and classification bias
7	Sample size and power	Evaluate whether the study had an adequate sample size to detect meaningful associations and whether an a priori power analysis was conducted and reported
8	A Priori Planning of Analyses	Determine whether the analysis plan was sufficiently described, whether subgroup and sensitivity analyses were described and reported
9	Statistical Methods & Confounding Control	Assess whether statistical methods were appropriate, key confounders were appropriately controlled, missing data for samples and genetic variants were handled correctly, and corrections for multiple testing were applied where necessary
10	Testing of assumptions and inferences for genetic analyses	Evaluate whether the assumptions underlying genetic analysis were tested and reported, such as relatedness and consistency with demographic characteristics
11	Appropriateness of inferences drawn from results	Determine whether the conclusions drawn by the authors are consistent, supported by the study findings, and appropriate methodology

For studies with control groups, scores of ≤ 35 indicate poor quality, scores between 36 and 45 indicate moderate quality, and scores greater than 45 represent good quality. Conversely, for studies without control groups, scores of ≤ 32 indicate poor quality, scores between 33 and 40 indicate moderate quality, and scores greater than 40 represent good quality.

## Results

### Study selection

**Fig. 1:**
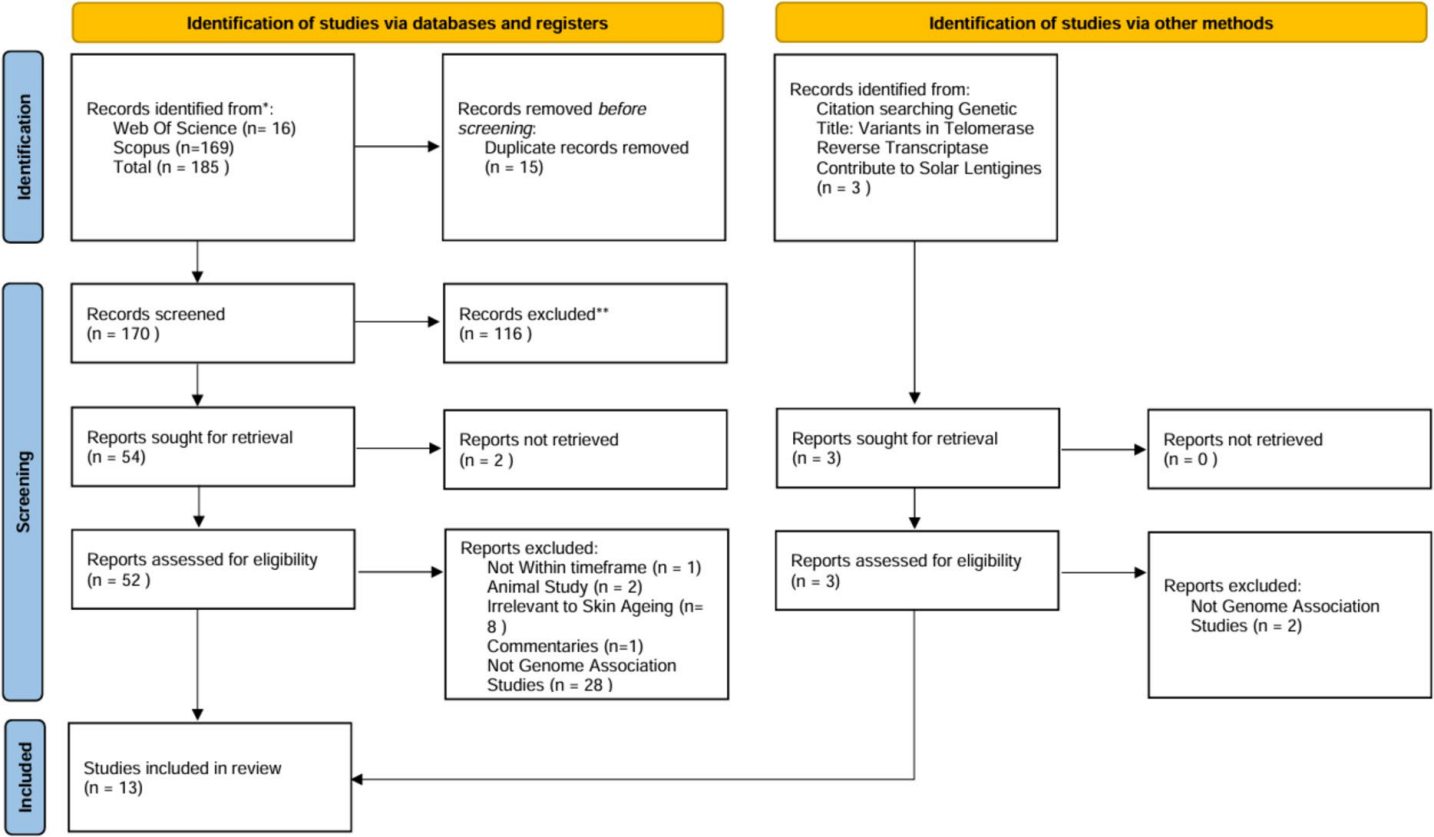
PRISMA Flow Diagram presenting the identification and selection process of articles. Source: Page et al. 2021

The systematic search of the online databases identified 185 papers. Following the removal of duplicates and screening for eligibility, 12 studies remained. One additional article was retrieved through the manual search of reference lists, leaving 13 articles to be included in this systematic review. Figure 1 highlights the identification and selection process in accordance with the PRISMA statement (Page et al. 2021).

## Data collection and extraction

A total of 13 articles were included in this study. Key data from each study were systematically extracted and organised for analysis. Findings from genome-wide association studies are summarised in Table 3, while results from candidate gene analyses are presented in Table 4.

**Table 3:** Genome-wide association studies with skin ageing phenotypes

Reference	Population Data	Gene (Polymorphism, allele)	Skin ageing phenotype	Result
(Chen et al. 2021)	Latin American 3377 females and 2877 males Mean age: 24.0 ± 5.3 years	*VAV3* rs2504460, G allele	Wrinkle PC1^*^	P-value: 1 × 10^–8^ β: 0.13
		*SLC45A2* rs173662, C allele		P-value: 3 × 10^–9^ β: 0.11
		*SLC45A2* rs185146		P-value: 1 × 10^–8^
		*IRF4* rs12203592, T allele		P-value: 2.8 × 10^–4^ β: 0.13
		*SPATA33* rs35063026, T allele		P-value: 2 × 10^–3^ β: 0.19
		rs147991442, A allele Intergenic region	Forehead wrinkles	P-value: 6 × 10^–10^ β:0.20
(Endo et al. 2018)	Japanese Females: 11,311	*PPARGC1B* rs251468, T allele	Age spots	P-value: 6.2 × 10^–12^ Odd ratio (OR): 0.77
		*RAB*_*11*_*FIP*_*2*_ rs61866017, T allele;		P-value: 9.7 × 10^–10^ OR: 0.78
		*RAB*_*11*_*FIP*_*2*_ rs35563099, T allele		P-value: 1.2 × 10^–6^ OR: 0.78
(Jacobs et al. 2014)	Dutch Population 2455 males, 3123 females Mean age: 67.1 years United Kingdom population: 110 males, 943 females Mean age: 53.1 years	*DLGAP1* rs11876749, C allele	Eyelid sagging	β: −0.14 P-value: 1.7 × 10^−8^ The C allele of the SNP rs11876749 showed the protective effect for moderate sagging eyelids (OR: 0.54; P-value: 6.1 × 10^−9^) in the recessive model
		*SMYD3* rs10924350, T allele		β: 0.10 P-value:1.4 × 10^−6^ T allele of rs10924350 exhibits risk effect on moderate eyelid sagging in the dominant model (OR:1.4; P-value: 1.6 × 10^–5^)
		*DLGAP1* rs4076011, T allele		β: −0.14 P-value: 2.7 × 10^–8^ T allele of rs4076011 showed protective effect for moderate eyelid sagging (OR:0.55; P-value: 1.4 × 10^−8^) in the recessive model
		*DLGAP1* rs8096287, G allele		β: −0.14 P-value: 2.1 × 10^–8^ G allele of rs8096287 showed protective effect for moderate eyelid sagging (OR:0.53; P-value: 3.8 × 10^−9^) in the recessive model
(Jacobs et al. 2015)	Dutch Population 1521 males and 1323 females Mean age: 66.9 ± 8.0 years	*IRF4* rs12203592, T allele	Facial pigmented spots	P-value: 1.9 × 10^−27^ % change in the pigmented spots area, per increase in effect allele (Δ per allele): 24.9%
		*SPATA33* rs35063026, T allele Exon 3 of c16orf55/SPATA33 ~ 250 kb upstream from *MC1R*		P-value: 9.4 × 10^−15^ Δ per allele: 20.29%
		*RALY* rs6059655, A allele Intron 8 of the *RALY* gene Located < 200 kb upstream from the skin colour gene *ASIP*		P-value: 2.6 × 10^−9^ Δ per allele: 14.6% LD between *ASIP* rs1205312 and rs6059655 was substantial (r^2^ = 0.59)
		rs62543565, C allele Intergenic Region, Located 30 kb upstream from BNC2 gene		The C allele showed the most significant association with facial-pigmented spots Found to be involved in skin colouration P-value: 2.3 × 10^−8^ Δ per allele: −6.4%
(Kim et al. 2021)	Korean population 1079 females Mean age: 40.81 ± 10.90 years	rs117381658, T allele Intergenic region Located < 500 kb downstream of *FCRL5*	Wrinkle	β: 0.952 ± 0.167 P-value: 1.52 × 10^–8^
		rs1961184, T allele Intergenic region Located < 500 kb downstream of *REEP3*		β: 0.840 ± 0.164 P-value: 3.60 × 10^–7^
		*LOC105373262 *rs1929013, G allele Located < 500 kb downstream of *ADSS*		β: −0.410 ± 0.092 P-value: 8.29 × 10^–6^
		*LOC107987094* rs7042102, T allele Located < 500 kb downstream of *SPTLC1*		β: 0.392 ± 0.088 P-value: 9.30 × 10^–6^
		*OCA2* rs74653330, T allele (Missense variant)	Pigmentation	β: −1.092 ± 0.189, P-value: 1.04 × 10^–8^
		rs34466224, A allele Intergenic region Located < 500 kb downstream of *NCLN*		β: 0.602 ± 0.124 P-value: 1.33 × 10^–6^
		*TNS1* rs11685354, A allele		β: −0.477 ± 0.099 P-value: 1.82 × 10^–6^
		*CDC42BPA* rs4653497, T allele (Missense variant)		β: 0.446 ± 0.098 P-value: 6.51 × 10^–6^
		*HS3ST4* rs59784607, T allele		β: −0.541 ± 0.121 P-value: 8.13 × 10^–6^
		rs76548385, T allele Intergenic region Located < 500 kb downstream of *UNCX*		β: 0.768 ± 0.173 P-value: 9.92 × 10^–6^
(Laville et al. 2016)	French Caucasian 501 females Mean age: 57.6 ± 6.4 years	*HLA-C* rs2853949, A allele	Facial solar lentigines	P-value: 6.24 × 10^–7^
		rs9350204, C allele Intergenic region	Forehead solar lentigines	P-value: 1.37 × 10^–8^
		rs9358294, G allele Intergenic region		P-value: 1.37 × 10^–8^
(Laville et al. 2019)	French Caucasian 502 females Mean age: 57.6 years	*H2AFY2* rs16927253, T allele	Upper eyelid sagging	OR (95%Cl): 0.25 (0.16–0.39) P-value: 1.72 × 10^–9^
		*H2AFY2* rs4746957, A allele		OR (95%Cl): 0.27 (0.15–0.49) P-value: 2.3 × 10^–8^
		*H2AFY2* rs2271699, G allele		OR (95%Cl): 0.43 (0.31–0.61) P-value: 6.97 × 10^–7^
		*H2AFY2* rs2394654, C allele		OR (95%Cl): 0.43 (0.31–0.61) P-value: 6.97 × 10^–7^
		*H2AFY2* rs3750770, T allele		OR (95%Cl): 0.42 (0.30–0.60) P-value: 8.85 × 10^–7^
		rs4109239, A allele Intergenic region Located ~ 15 kb away from *COL13A1*		OR (95%Cl): 0.42 (0.30–0.59) P-value: 5.85 × 10^–7^
		*ADAMTS18* rs12599182, A allele		OR (95%Cl): 0.44 (0.33–0.61) P-value: 3.48 × 10^–7^
(Park et al. 2021)	Korean population 128 women Less wrinkled (*n* = 51): 52.7 ± 7.0 years Wrinkled (*n* = 77): 56.3 ± 9.2 years	*EGFR* rs1861003, G allele	Wrinkle risk	OR (95%CI): 0.282 (0.083–0.952) P-value: 4.14 × 10^–4^
		*MMP16* rs6469206, G allele		OR (95%CI): 0.481 (0.244–0.949) P-value: 3.48 × 10^–2^
		*COL17A1* rs805698, T allele		OR (95%CI): 11.49 (2.225–76.08) *P*-value: 4.40 × 10^–3^
		*SOD3* rs4697073, A allele		OR (95%CI): 0.206 (0.045–0.949) *P*-value: 4.27 × 10^–2^
		*TNFRSF6B* rs1291206, A allele		OR (95%CI): 2.76 (1.34–5.66) P-value: 5.71 × 10^–2^
		*TNFRSF8* rs6690493, G allele		OR (95%CI): 0.189 (0.064–0.553) P-value: 2.38 × 10^–3^
		*NOS1* rs9658535, C allele		OR (95%CI): 3.81 (1.42–10.2) P-value: 7.74 × 10^–3^
(Peng et al. 2023)	TZL Cohort: Han Chinese 1059 males & 1905 females, Age: 31–86 years Mean age: 55.9 ± 9.4 years NSPT Cohort: Han Chinese 1109 males and 1845 females, Age: 19–83 years Mean age: 49.7 ± 13.2 years	*TERT* rs2853672, A allele	Non facial solar lentigines	Upregulate gene transcription TZL Cohort: β: 0.23 ± 0.02 P-value: 1.32 × 10^‒28^ NSPT Cohort β: 0.15 ± 0.02 P-value: 3.66 × 10^‒17^
		*TERT* rs2736098, T allele		Upregulate gene transcription TZL Cohort: β: −0.15 ± 0.02 P-value: 1.47 × 10^‒12^ NSPT Cohort β: −0.10 ± 0.02 P-value: 4.55 × 10^‒7^
		*TERT* rs2853669, G allele		Downregulate gene transcription TZL Cohort: β: 0.15 ± 0.01 P-value: 2.20 × 10^‒12^ NSPT Cohort β: 0.09 ± 0.02 P-value: 1.48 × 10^‒6^
		*TERT* rs2735940, A allele		Downregulate gene transcription TZL Cohort: β: −0.21 ± 0.02 P-value: 2.93 × 10^‒25^ NSPT Cohort Β: −0.15 ± 0.02 P-value: 5.35 × 10^‒17^
		*TERT* rs7712562, A allele		Downregulate gene transcription TZL Cohort: β: −0.23 ± 0.04 P-value: 8.37 × 10^‒11^ NSPT Cohort β: −0.17 ± 0.03 P-value: 1.56 × 10^‒8^
		*OCA2* rs1800414, T allele (Missense variant)	Non-facial solar lentigines	TZL Cohort: β: −0.21 ± 0.02 P-value: 2.93 × 10^‒25^ NSPT Cohort β: −0.15 ± 0.02 P-value: 5.35 × 10^‒17^
(Liu et al. 2019b)	Discovery Cohort: Hans Chinese 1534 females Mean age: 55.9 ± 9.3 years 31–86 years Second dataset: Hans Chinese 1084 females Mean age: 60.0 ± 5.5 years	*PEX3* rs3804540	Size of pigmented spots on forehead	P-value: 4.6 × 10^–9^ Effect: 0.60
		rs4962295 Intergenic region	Wrinkles under Eyes	P-value: 1.9 × 10^–8^ Effect: −0.26
		*SHC4* rs28392847	Crow’s feet	P-value:1.6 × 10^–8^ Effect size: −0.16
		*ARHGEF7* rs3825460	Size of pigmented Spots on cheek	P-value: 3.7 × 10^–8^ Effect size: 0.53
		*MYH11* rs76053540	Nasolabial fold	P-value: 5.0 × 10^–9^ Effect size: −0.27

Taizhou Longitudinal Cohort (TZL); National Survey of Physical Traits (NSPT); Linkage disequilibrium (LD)

*Overall wrinkling score for 4 wrinkling traits (wrinkles under eyes, glabellar—“frown lines”-, fine lateral canthal rhytids—“crow’s feet”- and fine forehead wrinkles)

**Table 4:** Candidate gene analysis with skin ageing phenotypes

Reference	Population Data	Gene/Polymorphism	Skin ageing phenotype	Result	
(Gao et al. 2017)	TZL cohort: Han Chinese 502 females Age: 32–85 years Mean age: 58 ± 10 years	*AHR* rs2066853, A allele (Missense variant)	Crow’s feet	AMR (95% Cl):1.07 (1.02–1.11) P-value: 2.1 × 10^–3^	
		*BNC2* rs10733310, T allele	Pigmented spots on the arms	GMR (95% Cl): 0.81 (0.71–0.91) P-value: 7.9 × 10^–5^	
		rs11979919, T allele Intergenic region, nearby gene: *COL1A2*	Laxity eyelids	GMR (95% Cl): 0.74 (0.60–0.89) P-value: 9.5 × 10^–5^	
		*OCA2* rs1800414, T allele (Missense variant)	Pigment back hand	GMR (95% Cl): 0.90 (0.82–0.99) P-value: 0.03	
		rs4886605, C allele Intergenic region, nearby gene: *CYP1A1*	Pigment back hand	GMR (95% Cl): 1.14 (1.04–1.25) P-value: 0.01	
		*SLC45A2* rs2672, T allele (Missense variant)	Pigment forehead	GMR (95% Cl): 1.15 (1.04–1.27) P-value: 0.01	
		*SOD2* rs4880, T allele (Missense variant)	Pigment forearm	GMR (95% Cl): 0.85 (0.74–0.99) P-value: 0.04	
		*MC1R* rs2228479, A allele (Missense variant)	Laxity cheeks	GMR (95% Cl): 1.06 (1.01–1.11) P-value: 0.03	
		*CYP1A1* rs4646421, A allele		GMR (95% Cl): 1.05 (1.01–1.09) P-value: 0.02	
		rs4886605, C allele Intergenic region, nearby gene: *CYP1A1*		GMR (95% Cl): 0.95 (0.92–0.99) P-value: 0.01	
(Laville et al. 2016)	French Caucasian 501 females Mean age: 57.6 ± 6.4 years	*MITF* rs17006579	Facial Solar Lentigines	P-value: 6.1 × 10^–4^ Q-value (FDR): 0.16	
		*MITF* rs7430957		P-value: 8.7 × 10^–4^ Q-value (FDR): 0.16	
		*MITF* rs9858495		P-value: 1.3 × 10^–3^ Q-value (FDR): 0.16	
		*MITF* rs1529585		P-value: 1.3 × 10^–3^ Q-value (FDR): 0.16	
		*MITF* rs1121878		P-value: 1.5 × 10^–3^ Q-value (FDR): 0.16	
		*MITF* rs576		P-value: 1.8 × 10^–3^ Q-value (FDR): 0.16	
		*MITF* rs1491687		P-value: 1.8 × 10^–3^ Q-value (FDR): 0.16	
		*MITF* rs6777363		P-value: 1.8 × 10^–3^ Q-value (FDR): 0.16	
		*MITF* rs704246		P-value: 2.0 × 10^–3^ Q-value (FDR): 0.16	
		*MITF* rs13071701		P-value: 2.3 × 10^–3^ Q-value (FDR): 0.16	
		*AMBP* rs7034903		P-value: 2.3 × 10^–3^ Q-value (FDR): 0.16	
		*SCEL* rs912292		P-value: 2.3 × 10^–3^ Q-value (FDR): 0.16	
		*OCA2* rs17650960		P-value: 2.8 × 10^–3^ Q-value (FDR): 0.18	
		*MITF* rs7623610		P-value: 3.2 × 10^–3^ Q-value (FDR): 0.19	
		*OCA2* rs1498519		P-value: 3.5 × 10^–3^ Q-value (FDR): 0.19	
		*SMPDL3A* rs6904500		P-value: 4.5 × 10^–3^ Q-value (FDR): 0.23	
		*MITF* rs1544978		P-value: 5.1 × 10^–3^ Q-value (FDR): 0.24	
		*AMBP* rs12377342		P-value: 5.2 × 10^–3^ Q-value (FDR): 0.24	
(Peng et al. 2023)	TZL Cohort: Han Chinese 1059 males & 1905 females, Mean age: 55.9 ± 9.4 years NSPT Cohort: Han Chinese 1109 males and 1845 females, Mean age: 49.7 ± 13.2 years SALIA: Caucasian 462 females Age: 66.7–79.4 years	*PPARGC1B* rs251468, T allele	Facial solar lentigines	β: −0.07 P-value: 4.63 × 10^–5^	
			Non-facial solar lentigines	β: −0.06 P-value: 8.49 × 10^–4^	
		rs61866017, T allele Intergenic region, nearby gene: *RAB*_*11*_*FIP*_*2*_		β: −0.07 P-value: 1.10 × 10^–3^	
		rs35563099, T allele Intergenic region, nearby gene: *RAB*_*11*_*FIP*_*2*_		β: −0.10 P-value: 4.38 × 10^–5^	
		rs10444039, A allele Intergenic region, nearby gene: *RAB*_*11*_*FIP*_*2*_		β: −0.09 P-value: 4.54 × 10^–5^	
		*HSPA*_*12*_*A* rs12259842, T allele		β: 0.08 P-value: 4.02 × 10^–6^	
		rs11198112, T allele Intergenic region, nearby gene: *RAB*_*11*_*FIP*_*2*_		β: −0.09 P-value: 7.62 × 10^–5^	
		*MFSD12* rs2240751, G allele (Missense variant)	Facial solar lentigines	β: −0.07 P-value: 4.83 × 10^–6^	
(Pardo et al. 2020)	Dutch Population 1793 Caucasian Age: > 45 years old	*IRF4* rs12203592	Pigmented spots	β: −0.111 ± 0.0102 P-value: 2.9 × 10^–27^	
		rs139810560 Intergenic region, nearby Genes: *DEF8, TUBB3, CENPBD1*		β: −0.056 ± 0.0105 P-value: 1.2 × 10^–7^	
		*SPATA33* rs35063026		β: −0.088 ± 0.0118 P-value: 1.4 × 10^–13^	
		rs35096708 Intergenic region, nearby gene: *ZNF276*		β: −0.040 ± 0.0069 P-value: 7.3 × 10^–9^	
		*MC1R* rs1805007		β: −0.081 ± 0.0119 P-value: 1.29 × 10^–11^	
		*RALY* rs6059655		β: −0.065 ± 0.0114 P-value: 1.61 × 10^–8^	
		rs12350739 Intergenic region, nearby genes: *BNC2, CNTLN*		β: 0.026 ± 0.0114 P-value: 2.02 × 10^–5^	
		*TYR* rs1042602		β: 0.024 ± 0.0062 P-value: 0.00014	
		rs478882 Intergenic region, nearby genes: *DOCK8-AS1, DOCK8, CBWD1*	Global wrinkling	β: −0.103 ± 0.0169 P-value: 1.19 × 10^–9^	
		rs10476781 Intergenic region, nearby genes: *NMUR2, CTB-12O2.1, LINC01470*		β: −0.145 ± 0.0361 P-value: 5.846 × 10^–5^	
(Rahmouni et al. 2022)	French Caucasian 570 middle-aged females	*XPC* rs2733537	Wrinkles	P-value: 0.00153783	Nucleotide excision repair pathway (FDR: 0.02, P-value: 2 × 10^–4^)
		*MNAT* rs4151374		P-value: 0.00519811	
		*DDB1* rs2230356		P-value: 0.00932987	
		*PSMB3* rs228275		P-value: 0.00035990	Proteasome Pathway (FDR: 0.04, P-value: 8 × 10^–4^)
		*PSMA1* rs11023246		P-value: 0.00118822	
		*PSMC2* rs10234363		P-value: 0.00182129	
		*PSMD2* rs34881379		P-value: 0.00257762	
		*PSMB5* rs12889177		P-value: 0.00448658	
		*PSMD4* rs7489		P-value: 0.00741838	
		*RAD23B* rs16912372	Sagging	P-value: 0.00054012	Nucleotide excision repair pathway (FDR: 0.05, P-value: 2 × 10^–4^)
		*DDB1* rs2230356		P-value: 0.00067031	
		*ERCC8* rs3117		P-value: 0.00558406	
		*POLE4* rs12713820		P-value: 0.00670101	
		*RPS6KA2* rs9347129		P-value: 0.00017583	mTOR signalling pathway (FDR: 0.05, P-value: 8 × 10^–4^)
		*PIK3R1* rs251406		P-value: 0.00194587	
		*TSC1* rs7040593		P-value: 0.00236903	
		*CAB39L* rs2407616		P-value: 0.00262206	
		*PIK3CA* rs1607237		P-value: 0.00282556	
		*RPTOR* rs2048753		P-value: 0.00316581	
		*VEGFA* rs1570360		P-value: 0.00717622	
		*PMM2* rs17747560		P-value: 0.00167131	Amino sugar and nucleotide sugar metabolism (FDR: 0.05, P-value: 2.2 × 10^–4^)
		*GFPT2* rs6893651		P-value: 0.00261209	
		*GMDS* rs3778565		P-value: 0.00418288	
		*CYB5R1* rs4989513		P-value: 0.00618583	
		*CMAS* rs728034		P-value: 0.00702727	

Arithmetic Mean Ratio (AMR); Geometric Mean Ratio (GMR); False Discovery Rate (FDR); Taizhou Longitudinal Cohort (TZL); National Survey of Physical Traits (NSPT)

## Quality assessment

Table 5 presents the Q-Genie quality appraisal results for the included studies, with all articles meeting the high-quality threshold (scores ≥ 45/77). While methodological rigour was consistently strong (Items 1, 2, 4–6, 8–9), some studies lacked justification for sample sizes (Item 3) or detailed statistical reporting (Item 7). Notably, Jacobs et al. (2014) achieved the highest score (58), exemplifying excellence in methodological coherence and comprehensive reporting. These results collectively support the reliability of the evidence base for subsequent analysis.

**Table 5:** Q-Genie quality assessment scores for the included studies

Studies	Items											Total Score
	1	2	3	4	5	6	7	8	9	10	11	
(Peng et al. 2023)	6	5	–	5	6	5	4	6	6	5	6	54
(Gao et al. 2017)	7	5	–	5	3	4	4	5	7	3	6	49
(Jacobs et al. 2015)	7	6	–	5	5	7	5	5	5	5	7	57
(Jacobs et al. 2014)	6	5	–	4	5	5	5	6	6	5	6	58
(Liu et al. 2019b)	6	6	–	6	5	5	3	5	6	5	6	53
(Laville et al. 2016)	6	5	–	5	5	5	5	5	6	3	6	51
(Laville et al. 2019)	6	6	–	5	5	5	5	5	7	5	6	55
(Chen et al. 2021)	6	5	–	4	5	5	5	5	7	5	6	53
(Kim et al. 2021)	7	6	–	5	5	5	5	5	4	5	6	53
(Pardo et al. 2020)	6	6	–	6	5	6	3	3	6	6	4	51
(Park et al. 2021)	6	5	5	6	5	3	7	4	4	4	5	54
(Rahmouni et al. 2022)	6	4	–	5	5	3	5	6	6	4	5	49
(Endo et al. 2018)	6	5	5	4	4	3	4	5	5	5	6	52

## Synthesis of results

A total of 37,697 participants were included across the 13 studies reviewed. Of these, 27,387 (72.65%) were female and 8,522 (22.60%) were male. One study (Pardo et al. 2020) did not report the gender distribution of its participants. Overall, the data reflect a predominant focus on female populations. The studies encompassed diverse ethnic groups, with six studies involving European participants, six focusing on Asian populations, and one including Latin American participants.

In terms of phenotype focus, four studies reported skin sagging, seven addressed wrinkles, and eight investigated hyperpigmentation. Tables 3 and 4 present a detailed extraction of results for each study. Collectively, the studies analysed 115 single nucleotide polymorphisms (SNPs) across 99 distinct genes, offering valuable insights into the genetic basis of various skin ageing phenotypes.

The Oculocutaneous Albinism II (OCA2) gene emerged as the most frequently studied, appearing in four articles and consistently showing an association with hyperpigmentation. The next most commonly investigated genes were Interferon Regulatory Factor 4 (IRF4) and Melanocortin 1 Receptor (MC1R), both linked to the development of wrinkles and facial pigmented spots. To improve clarity and ease of interpretation, the synthesis of findings is stratified by specific skin ageing phenotypes, such as wrinkles, skin sagging, and hyperpigmentation, and further categorised by population groups.

## Wrinkles

Four SNPs in the Han Chinese population have been identified as significantly associated with wrinkles. The missense variant rs2066853 in the *AHR* gene, with the A allele as the effect allele, increases the risk of crow’s feet by 7% per allele copy. Conversely, rs4962295 (intergenic region), *MYH11* rs76053540, and *SHC4* rs28392847 exhibit protective effects against wrinkles, as evidenced by their negative effect size values.

In the Korean population, 11 single nucleotide polymorphisms (SNPs) have been identified as significantly associated with wrinkle formation, five exhibiting protective effects and six linked to increased risk. The protective variants include the G allele of LOC105373262 rs1929013 (near the *ADSS2* gene), the G allele of EGFR rs1861003, the G allele of *MMP16* rs6469206, the G allele of *TNFRSF8* rs6690493, and the A allele of *SOD3* rs4697073. Among these, the G allele of *TNFRSF8* rs6690493 demonstrated the most pronounced protective effect, reducing the risk of wrinkles by 81.1%.

Conversely, six SNPs were associated with an increased risk of wrinkle formation. These include the T allele of COL17A1 rs805698, two intergenic variants, the T allele of rs117381658 (near FCRL5) and the T allele of rs1961184 (near REEP3), the C allele of NOS1 rs9658535, the T allele of LOC107987094 rs7042102 (near SPTLC1), and the A allele of TNFRSF6B rs1291206. Among these, the T allele of the missense variant COL17A1 rs805698 showed the strongest risk association, increasing the likelihood of wrinkle development by 11.49-fold.

In the French Caucasian population, wrinkle development was significantly linked to the nucleotide excision repair (NER) and proteasome pathways, with several top-ranked genes showing associations at a false discovery rate (FDR) of less than 0.05. Key genes within the NER pathway included *DDB1* rs2230356, *MNAT1* rs4151374, and *XPC* rs2733537. In the proteasome pathway, significant variants were identified in *PSMA1* rs11023246, *PSMB3* rs228275, *PSMB5* rs12889177, *PSMC2* rs10234363, *PSMD2* rs34881379, and *PSMD4* rs7489. In contrast, data from the Dutch population revealed that two intergenic variants, rs10476781 and rs478882, were associated with a reduced risk of wrinkle formation, as indicated by their negative beta values.

In the Latin American population, five single nucleotide polymorphisms (SNPs) were identified as conferring increased risk for wrinkle formation, as indicated by their positive beta values. These included an intergenic variant (A allele of rs147991442), as well as the T allele of *IRF4* rs12203592, the C allele of *SLC45A2* rs173662, the T allele of *SPATA33* rs35063026, and the G allele of *VAV3* rs2504460. Additionally, another variant, *SLC45A2* rs185146, was significantly associated with wrinkles, although the direction of its effect on wrinkle development was not specified.

## Skin sagging

In the Han Chinese population, four SNPs were significantly associated with skin sagging. Protective intergenic regions, the C allele of rs4886605 (nearby gene: *CYP1A1*) and the T allele of rs11979919 (Nearby gene: *COL1A2*) reduced cheek laxity by 5% and eyelid laxity by 26% per allele copy, respectively. In contrast, the A allele of *CYP1A1* rs4646421 and the A allele of the missense variant of *MC1R* rs2228479 demonstrated additive effects by increasing cheek laxity by 5 and 6% per allele copy, respectively.

In the French Caucasian population, skin sagging was significantly associated with the NER pathway, the amino sugar and nucleic acid metabolism pathway, and the mammalian target of rapamycin (mTOR) signalling pathway, as evidenced by top-ranked genes with a False Discovery Rate (FDR) < 0.05. Specifically, *DDB1* rs2230356, *ERCC8* rs3117, *POLE4* rs12713820, and *RAD23B* rs16912372 were associated with the NER pathway. Meanwhile, *CMAS* rs728034, *CYB5R1* rs4989513, *GFPT2* rs6893651, *GMDS* rs3778565, and *PMM2* rs17747560 were the key genes in the amino sugar and nucleic acid metabolism pathway. Additionally, *CAB39L* rs2407616, *PIK3CA* rs1607237, *PIK3R1* rs251406, *RPS6KA2* rs9347129, *RPTOR* rs2048753, *TSC1* rs7040593, and *VEGFA* rs1570360 were involved in the mTOR signalling pathway.

In addition to these pathways, three other genes were significantly associated with eyelid sagging and exhibited a protective effect against skin sagging. Notably, the A allele of *ADAMTS*18 rs1259918 and a protective intergenic region near *COL13A1* (A allele of rs4109239) reduced skin sagging by 56% and 58% per allele copy, respectively. Furthermore, five SNPs of *H2AFY2* (T allele of rs16927253, A allele of rs4746957, G allele of rs2271699, C allele of rs2394654 and T allele of rs3750770) also demonstrated protective effects, reducing skin sagging by 57 to 75%, with the T allele of rs16927253 showing the strongest protective effect at 75%.

In the Dutch and United Kingdom populations, two genes were significantly associated with eyelid sagging. Variants of *DLGAP1* (T allele of rs4076011, G allele of rs8096287, C allele of rs11876749) provided a protective effect against moderate eyelid sagging, as indicated by their negative beta values and odds ratio. Conversely, the T allele of *SMYD3* rs10924350 was associated with an increased risk of moderate eyelid sagging, as evidenced by its positive beta value and odds ratio.

## Hyperpigmentation

Seven genes were significantly associated with pigmented spots in the Han Chinese population. Three genes, the T allele of *BNC2* rs10733310, a missense variant of the G allele of *SOD2* rs4880, and a missense variant of the T allele of *OCA2* rs1800414, exhibited protective effects. These variants reduced pigmented spots by 10 to 19%, with the T allele of rs10733310 demonstrating the strongest effect, as evidenced by their Geometric Mean Ratio (GMR).

Conversely, four genes were associated with an increased risk of pigmented spots, including one intergenic region near *CYP1A1* (C allele of rs4886605), a missense variant of the T allele of *SLC45A2* rs26722, *ARHGEF7* rs3825460, and *PEX3* rs3804540. The additive risk effects of *CYP1A1* and *SLC45A2* were supported by their GMR values. For instance, each copy of the C allele of rs4886605 increases pigmented spots by 14%, while each copy of the T allele of rs26722 increases it by 15%. Moreover, the risk effects of *ARHGEF7* and *PEX3* were supported by their positive effect sizes.

Furthermore, six genes and twelve SNPs were found significantly associated with solar lentigines (SLs). Among them, only three SNPs, the T allele of *HSPA12A* rs12259842, the G allele of *TERT* rs2853669, and the A allele of *TERT* rs2853672, exhibited a risk effect towards SL. Conversely, ten SNPs across four genes exhibited protective effects against SLs. These include three intergenic regions near *RAB*_*11*_*FIP*_*2*_ (T allele of rs35563099, A allele of rs10444039, and T allele of rs61866017), three variants of *TERT* (A allele of rs7712562, A allele of rs2735940, and T allele of rs2736098), a missense variant of T allele of *OCA2* rs1800414, the G allele of *MFSD12* rs2240751, and the T allele of *PPARGC1B* rs251468. These protective effects were supported by their negative beta values.

In the Korean population, six genes were significantly associated with pigmentation. Among these, three genes, including a missense variant of the T allele of *OCA2* rs74653330, the T allele of *HS3ST4* rs59784607, and the A allele of *TNS1* rs11685354, exhibited a protective effect on pigmentation, as evidenced by their negative beta values. Conversely, the other three genes, the missense variant of the T allele of *CDC42BPA* rs4653497 and two intergenic regions (A allele of rs34466224, nearby gene: *NCLN*; T allele of rs76548385, nearby gene: *UNCX*), were associated with increased pigmentation, as indicated by their positive beta values.

In the Japanese population, two genes encompassing three SNPs were significantly associated with age spots and exhibited protective effects. These include the T allele of *PPARGC1B* rs251468, which reduced the age spots by 23%, and two intergenic regions near *RAB*_*11*_*FIP*_*2*_ (T allele of rs61866017 and T allele of rs35563099), each reducing the risk by 22%.

In the Dutch population, twelve genes across nine SNPs were significantly associated with pigmented spots. Among these, *IRF4* rs12203592, *MC1R* rs1805007, *RALY* rs6059655, and three intergenic regions (rs139810560, nearby genes: *BNC2*, *CNTLN*; rs35096708, nearby gene: *ZNF276*; C allele of rs62543565, nearby gene: *BNC2*) exhibited a protective effect against pigmented spots, as indicated by their negative beta values and negative effect sizes. Conversely, *TYR* rs1042602 and an intergenic region near BNC2 and CNTLN (rs12350739) exhibited a risk effect on pigmented spots, as evidenced by their positive beta values. In addition, the T allele of *IRF4* rs12203592 and *SPATA33* rs35063026, along with the A allele of *RALY* rs6059655, demonstrated a risk effect for facial pigmented spots, indicated by positive effect sizes.

In the French Caucasian population, there were seven genes and 21 SNPs that were significantly associated with SLs, as all of them passed the FDR threshold (< 0.25). Notably, two intergenic regions (C allele of rs9350204, G allele of rs9358294) were associated with forehead SLs, In contrast, the remaining genes were associated with facial SLs, including *AMBP* rs7034903, *AMBP* rs12377342, *FABP7* rs6904500 (nearby gene: *SMPDL3A*), A allele of *HLA-C* rs2853949, multiple variants of *MITF* (rs17006579, rs7430957, rs9858495, rs1529585, rs1121878, rs576, rs1491687, rs6777363, rs704246, rs13071701, rs7623610, rs1544978), *OCA2* rs17650960, *OCA2* rs1498519 and *SCEL* rs912292.

## Discussion

### Wrinkles

Wrinkle formation is a multifactorial process influenced by both intrinsic genetic mechanisms and extrinsic environmental factors. One key player is the NOS1 gene, which encodes nitric oxide synthase-1. This enzyme catalyses the production of nitric oxide (NO), a reactive free radical that indirectly activates matrix metalloproteinase-13 (MMP-13) (Zaragoza et al. 2002; Man et al. 2022). MMP-13 contributes to the degradation of collagen types I and III, two essential components of the dermal extracellular matrix (ECM), ultimately leading to the structural weakening of the dermis and the appearance of wrinkles (Zaragoza et al. 2002; Feng et al. 2024).

Additionally, the aryl hydrocarbon receptor (AHR) plays a pivotal role in mediating environmental damage to the skin. AHR acts as a cellular sensor for environmental pollutants such as polycyclic aromatic hydrocarbons (PAHs) and particulate matter (PM). Upon binding to these pollutants, AHR translocates to the nucleus, where it dimerises with the aryl hydrocarbon receptor nuclear translocator (ARNT). This AHR-ARNT complex then binds to xenobiotic-responsive elements (XREs) in DNA, initiating the transcription of detoxification enzymes, notably cytochrome P450 1A1 (CYP1A1) (Parrado et al. 2019). While CYP1A1 plays a protective role in metabolising xenobiotics, its enzymatic activity also generates reactive oxygen species (ROS) such as hydrogen peroxide as a byproduct (Grishanova and Perepechaeva 2022). These ROS further upregulate the expression of matrix metalloproteinases (MMPs), particularly MMP-1, which degrades collagen and other structural proteins in the ECM (Qiao et al. 2017), contributing to the formation of crow’s feet and other visible signs of skin ageing.

Beyond MMPs, *COL17A1*, which encodes collagen XVIIA1, a crucial component of hemidesmosomes at the dermal–epidermal junction (DEJ), plays an essential role in maintaining the structural integrity and mechanical strength of the epidermis. A missense variant in *COL17A1* compromises the stem cell niche at the DEJ and destabilises hemidesmosomes. This impairs keratinocyte differentiation and adhesion, leading to epidermal thinning and flattening of the rete ridges. Together, these changes reduce skin resilience and promote wrinkle formation (Xiang et al. 2022). Interestingly, the *VAV3* gene induces *COL17A1* expression, contributing to skin resistance against mechanical stress (Liu et al. 2019a). However, a specific polymorphism, rs2504460, located near the transcription start site of *VAV3.1*, a truncated version of *VAV3*, lacks several conserved protein domains, potentially resulting in reduced *COL17A1* expression and accelerating skin ageing (Chen et al. 2021). This warrants further investigation into the precise role of rs2504460 in wrinkle progression.

Melanogenesis also significantly influences wrinkle development. For example, *IRF4*, which encodes interferon regulatory factor 4, supports melanin synthesis by upregulating tyrosinase expression through interactions with the microphthalmia-associated transcription factor (*MITF*) (Pośpiech et al. 2020). The T allele of rs12203592 reduces *IRF4* binding, thereby impairing melanin production (Visser et al. 2015) and increasing sunlight sensitivity (Hernando et al. 2018), potentially accelerating UV-induced wrinkling. Furthermore, the *SPATA33* rs35063026, located 250 kb upstream of *MC1R* (melanocortin 1 receptor), might indirectly influence *MC1R* in regulating eumelanin and pheomelanin production. Impaired *MC1R* function skews the balance towards pheomelanin, which provides weaker UV shielding than eumelanin (Liu et al. 2016). Consequently, increased UV exposure upregulates MMP expression, leading to collagen degradation within the ECM (Feng et al. 2024). Despite these findings, additional studies are necessary to uncover the regulatory mechanisms linking *SPATA33* and *MC1R*.

Interestingly, the *SLC45A2* gene, which encodes a membrane-associated transporter for maintaining neutral pH in melanosomes, facilitates tyrosinase activity during melanogenesis (Linh et al. 2020). Although melanogenesis offers UV protection, the C allele of rs173662 paradoxically correlates with increased wrinkling. This suggests the need for further research into the underlying mechanisms of this polymorphism.

Additional genetic contributors include *REEP3* and *SPTLC1*, both of which are associated with moisture retention in the skin. *REEP3*, encoding receptor expression-enhancing protein 3, promotes adiponectin production (Huang et al. 2021), enhancing hyaluronic acid synthesis via the AMPK/PPARα pathway. Hyaluronic acid is essential for retaining skin moisture and preventing moisture loss (Yamane et al. 2011). Similarly, *SPTLC1*, a key gene for ceramide biosynthesis, supports epidermal hydration by producing ceramides through serine palmitoyl transferase activity (Kleuser and Japtok 2013; Rabionet et al. 2014). Despite their protective roles, polymorphisms such as *REEP3* rs11961184 and *SPTLC1* rs7042102 are associated with increased wrinkling. These associations highlight the need for further investigation into their specific mechanisms.

In addition, research on the French population has linked the wrinkle phenotype with pathways like nuclear excision repair (NER) (P: 2 × 10^–4^, FDR: 0.02) and proteasome pathway (P: 8 × 10^–4^, FDR: 0.04) (Rahmouni et al. 2022). NER, a critical DNA repair mechanism, removes bulky lesions caused by UV exposure, such as cyclobutane pyrimidine dimers (CPDs) and 6–4 photoproducts (6-4PPs). However, NER activity declines with age in dermal fibroblasts (Tigges et al. 2014), leading to inadequate photolesion repair and reduced collagen synthesis (Tigges et al. 2014), which collectively promote wrinkle formation. Further research is needed to clarify the mechanism of NER in wrinkle formation. Similarly, the proteasome activity is essential for degrading damaged and abnormal proteins and maintaining cellular function. However, its activity declines with age, as seen in dermal fibroblasts, due to reduced proteasome subunits and mRNA expression (e.g., *PSMA1*, *PSMB5*). This decline leads to the accumulation of oxidized proteins, promoting cellular senescence (Hwang et al. 2007). Further studies are needed to clarify the impact of impaired proteasome function on skin wrinkling.

While many genes contribute to wrinkle formation, some exhibit protective effects. For instance, *EGFR* encodes epidermal growth factor receptor, which interacts with epidermal growth factor (EGF) to enhance collagen expression and inhibit MMP activity (Romana-Souza et al. 2019). Similarly, *ADSS2* encodes adenylosuccinate synthetase isozyme 2 to promote adenosine synthesis, which stimulates the production of collagen types I and III by activating adenosine A2A receptors (Marucci et al. 2022).

In contrast, while *MMP16* encodes matrix metalloproteinase 16 and is involved in type I collagen degradation (Feng et al. 2024), its lower efficiency than other MMPs may reduce its contribution to wrinkle formation. Instead, its primary association with malignancy (Al-Atif 2022) could explain its protective role in wrinkles. Finally, *SOD3* encodes extracellular Superoxide Dismutase, which promotes collagen synthesis by activating AMPK and Nrf2/HO-1 pathways and inhibits MMP production. Additionally, *SOD3* could protect type I and IV collagens from oxidative damage (Lee et al. 2021b).

## Skin sagging

The formation of skin sagging results from a complex interplay of genetic and environmental mechanisms. For example, *SMYD3*, which encodes SET and MYND domain-containing protein 3, binds to the MMP-9 promoter and induces its expression (Cock-Rada et al. 2012). Since MMP-9 is a gelatinase that degrades collagen and elastic fibres, its activity directly impacts the extracellular matrix (ECM) of the eyelid, contributing to visible sagging (Damasceno et al. 2015). Similarly, a missense variant of *MC1R* (rs2228479, A allele) may increase pheomelanin production due to diminished receptor activity (Liu et al. 2016), which could eventually contribute to cheek laxity due to increased UV exposure. However, further investigation is needed to clarify the role of *MC1R* variants in melanogenesis and their specific impact on cheek laxity.

Additionally, environmental factors play a significant role. For instance, *CYP1A1*, a member of the cytochrome P450 enzyme family, is upregulated when environmental pollutants activate *AHR* (Parrado et al. 2019; Kyoreva et al. 2021). Increased *CYP1A1* expression generates ROS, such as hydrogen peroxide, which induces MMP-1 to degrade collagen in ECM (Brenneisen et al. 1997), exacerbating cheek laxity. Interestingly, an intergenic polymorphism near *CYP1A1* (rs4886605, C allele) appears to confer a protective effect against cheek laxity. This finding underscores the need for further research to unravel the regulatory mechanisms of this polymorphism in skin sagging.

Although *AHR* and *CYP1A1* share a similar mechanism involving collagen degradation, their distinct associations with specific skin ageing phenotypes, *AHR* with wrinkles and *CYP1A1* with cheek laxity (skin sagging), remain unclear. Currently, there is no literature providing a definitive explanation for why these genes are not simultaneously linked to both wrinkles and skin sagging. Since skin sagging results from the combined degradation of collagen and elastic fibres, further studies are warranted to uncover the potential mechanisms of *CYP1A1* in skin sagging. These investigations could focus on exploring its role in elastin degradation, particularly through the regulation of MMPs, such as MMP-12 and MMP-7, which predominantly target elastin (Feng et al. 2024).

In addition, research on the French population has identified pathways associated with skin sagging, including nuclear excision repair (NER) (P: 2 × 10^–4^, FDR: 0.05), amino sugar and nucleotide sugar metabolism (P: 2.2 × 10^–4^, FDR: 0.05) and mTOR signalling pathway (P: 8 × 10^–4^, FDR: 0.05) (Rahmouni et al. 2022). The role of NER in skin sagging is similar to its involvement in wrinkle formation, where impaired DNA repair mechanisms contribute to cellular senescence. While the biological role of amino sugar and nucleotide sugar metabolism in skin sagging remains unclear, glycation processes involving advanced glycation end products (AGEs) play a critical role. AGEs induce cross-linking of collagen fibres, reducing skin elasticity and flexibility (Lee et al. 2021a). Moreover, AGEs promote fibroblast senescence, impairing collagen and elastin synthesis, which further contributes to skin sagging (Zheng et al. 2022).

Similarly, although there is no direct study on the mTOR signalling pathway in skin sagging; however, it plays a pivotal role in ageing. The mammalian target of rapamycin complex 1 (mTORC1), a major regulator of autophagy, becomes hyperactivated in aged fibroblasts (Carroll et al. 2017). This hyperactivation inhibits autophagy (Yu et al. 2010), leading to decreased synthesis of collagen, elastin and hyaluronan, while increasing expression of MMP-1. These changes collectively reduce dermal stiffness, promoting the development of sagging skin (Tashiro et al. 2014).

While many genes contribute to skin sagging formation, some exhibit protective effects. For instance, *COL1A2* encodes the α2(I) chains of type I collagen (Makareeva and Leikin 2014), which constitutes 80–85% of the dermal ECM (Davison-Kotler et al. 2019). The ECM plays a crucial role in maintaining skin resilience, elasticity, and mechanical strength (Damasceno et al. 2015), all of which are essential for preventing eyelid laxity. Similarly, *COL13A1,* which encodes the alpha chain of type XIII collagen ​(Laville et al. 2019), is integral to cell–cell and cell–matrix junctions. By stabilising adherens junctions, *COL13A1* helps to preserve epidermal architecture and prevent skin sagging. Although direct studies on *COL13A1* and eyelid sagging are limited, its colocalization with E-cadherin in adherens junctions suggests a role in maintaining keratinocyte connections and epidermal integrity (Peltonen et al. 1999).

Finally, *ADAMTS18*, a member of the ADAMTS family of metalloproteinases, plays a critical role in ECM assembly and degradation (Nie and Zhang 2023). While its specific role in skin tissue and eyelid sagging is not fully understood, further research is warranted to explore its protective potential.

## Hyperpigmentation

The formation of age spots or SL results from a complex interplay of genetic and environmental mechanisms. For instance, previous sections (Wrinkles) discussed *SLC45A2* and *TYR* (tyrosinase), which play roles in melanogenesis. In addition, *PEX3*, which encodes an integral peroxisomal membrane protein (Waterham and Ebberink 2012), is vital in peroxisome metabolism. Peroxisomal metabolism may promote melanin production in melanocytes, as indicated by elevated catalase levels, an antioxidant of peroxisome metabolism (Maresca et al. 2008). Therefore, further research is needed to clarify the role of *PEX3* in melanogenesis. Furthermore, *CYP1A1*-induced ROS not only upregulates the expression of MMPs but also stimulates the production of α-melanocyte-stimulating hormone (α-MSH), which is involved in melanogenesis (Rousseau et al. 2007; Yuan and Jin 2018; Peng et al. 2019). These findings could explain their role in pigmented spots on the forehead.

Some genes or SNPs also exhibit protective effects against hyperpigmentation. For instance, the *OCA2* gene encodes the “P protein”, an integral melanosomal membrane protein crucial for regulating the pH of melanosomes, which is essential for melanin synthesis (Park et al. 2015). Missense variants (T allele of rs18000414 and T allele of rs74653330) may disrupt its activity, leading to increased melanosome acidification. This occurrence could hinder melanogenesis and possibly protect against SLs.

In addition, *SOD2* encodes mitochondrial superoxide dismutase 2, which is a mitochondrial antioxidant (Treiber et al. 2012). A missense variant of the G allele of *SOD2* rs4880 may cause excessive ROS accumulation, leading to mitochondrial fission. This fragmentation activates the ERK pathway, causing phosphorylation and proteasomal degradation of MITF, ultimately reducing melanin synthesis and the risk of pigmented spots on the forearm (Kim et al. 2014).

Additionally, *TUBB3*, a nearby gene in the intergenic region (rs139810560), encodes tubulin-β-III, forming MC1R-TUBB3 chimeric receptors that desensitize melanocytes to α-MSH signalling (Dalziel et al. 2011), thereby preventing excessive melanogenesis that could cause pigmented spots. Further studies are warranted to elucidate the regulatory mechanism between the intergenic region and *TUBB3*.

Furthermore, *MFSD12* encodes a transmembrane protein that influences pheomelanin synthesis via cysteine transport into melanosomes. A missense variant of G allele of MFSD12 rs2240751 may impair this function, reducing cysteine import and shifting melanin production toward eumelanin, resulting in darker pigmentation (Adelmann et al. 2020). Interestingly, the negative beta value of rs2240751 (−0.07) suggests a protective effect against facial SLs. However, this apparent discrepancy between its role in pigmentation and its protective effect on SLs highlights the need for further research to clarify the underlying mechanisms.

Moreover, *PPARGC1B* encodes Peroxisome proliferator-activated receptor-γ (PPAR-γ) coactivator 1β (PGC-1β), which directly induces the expression of the melanocyte-specific form of MITF (MITF-M) that mediates tyrosinase induction and melanogenesis in melanocytes (Shoag et al. 2013). However, current research does not yet explain how the T allele of *PPARGC1B* rs251468 contributes to the protection against age spots and SLs.

Additionally, *RAB*_*11*_*FIP*_*2*_, encoding Rab_11_-FIP_2_, regulates vesicular transport, including melanosome trafficking to the plasma membrane (Endo et al. 2018). Although direct research on its role in age spots and SLs is lacking, related studies reported that Rab_11b_ could prevent melanosome aggregation by aiding the trafficking of mature melanosomes to the plasma membrane for secretion (Tarafder et al. 2014). Further investigation is needed to confirm its protective effect in age spots and SLs.

Furthermore, the *TERT* gene, which encodes telomerase reverse transcriptase, has been implicated in SLs. Upregulation of *TERT* is reportedly linked to SLs, with specific alleles showing differential effects on gene transcription. Among these, rs2853672 and rs2736098 are associated with increased *TERT* transcription, while rs2853669, rs2735940, and rs7712562 are linked to decreased transcription. Interestingly, the T allele of rs2736098, which upregulates *TERT* transcription, exhibits a negative beta value (β: −0.15 in the TZL cohort and β: −0.10 in the NSPT cohort), suggesting a protective effect against non-facial SLs. In contrast, the G allele of rs2853669, associated with downregulation of *TERT* transcription, shows a positive beta value (β: 0.15 in the TZL cohort and β: 0.09 in the NSPT cohort), indicating a risk effect toward non-facial SLs (Peng et al. 2023). This apparent discrepancy between transcriptional activity and protective or risk effects warrants further investigation to clarify the underlying mechanisms.

Interestingly, polymorphisms in melanogenesis genes may have both protective and detrimental effects on hyperpigmentation. For instance, the missense variant of *MC1R* rs1805007 may encode a dysfunctional receptor that decreases eumelanin production, resulting in fewer pigmented spots (Sherry et al. 2001). However, further studies are needed to confirm its protective role. Additionally, *BNC2* is suggested to promote the development and survival of melanocytes by inducing the expression of pigmentation genes such as *KITLG* and *CSF1* (Patterson and Parichy 2013). This may explain the detrimental effects of the intergenic region near *BNC2* (rs12350739) on pigmented spots, but not the protective effects of the T allele of *BNC2* rs10733310 and the intergenic region near *BNC2* (C allele of rs62543565).

Besides, genetic variants near the MC1R locus exhibit complex associations with skin pigmentation. In the Dutch population, (Pardo et al. 2020) reported that *SPATA33* (rs3506302), located 250 kb upstream of *MC1R* (Liu et al. 2016), exerted a protective effect against pigmented spots (β =  − 0.088), suggesting a role in reduced pigmentation. Intriguingly, this contrasts with findings by (Jacobs et al. 2015), where the T allele of rs3506302 was associated with a 20.29% increased risk of facial pigmented spots per allele, implying divergent population-specific or context-dependent effects. Similarly, the *IRF4* polymorphism rs12203592, which reduces IRF4 binding affinity and impairs melanin production (Visser et al. 2015), showed protective effects in (Pardo et al. 2020) (β =  − 0.111) but was linked to a 24.9% elevated risk per T allele in (Jacobs et al. 2015). These opposing associations highlight the need to consider genetic background, environmental interactions, or methodological differences when interpreting pigmentation-related variants.

The *RALY* gene variant rs6059655, situated within 200 kb upstream of *ASIP*, regulates melanogenesis by potentially influencing production of agouti signalling protein (ASIP), which competitively inhibits α-MSH binding to *MC1R*. Strong linkage disequilibrium between rs6059655 and *ASIP* rs1205312 (r^2^ = 0.59) supports long-range regulatory effects on *ASIP* expression (Jacobs et al. 2015). However, contradictory associations emerge across studies: (Pardo et al. 2020) reported that rs6059655 conferred protection against pigmented spots (β =  − 0.065), whereas (Jacobs et al. 2015) observed that the A allele increased facial pigmented spot risk by 24.9% per allele. This discrepancy may reflect differences in reference allele designation between studies, emphasising the need for standardised reporting.

Intriguingly, in Latin American populations, the T alleles of *SPATA33* rs35063026 and *IRF4* rs12203592 demonstrate apparent pleiotropy, associating with both increased pigmentation and enhanced wrinkle formation. These dual effects likely arise through independent pathways influencing melanin production and dermal integrity, with population-specific environmental factors (e.g., UV exposure) further modulating phenotypic outcomes. When skin is exposed to UV radiation, keratinocytes will absorb UV light and increase production of α-MSH via activation of p53-mediated propiomelanocortin (POMC) promoter. The secreted α-MSH subsequently binds to MC1R on melanocytes, which in turn stimulates melanogenesis (Maddodi et al. 2012). The T allele of IRF4 rs12203592 is known to be associated with fairer skin colour and reduced tanning capacity (Zhang et al. 2013). In individuals with lighter skin, the melanogenesis response may be uneven, leading to localised areas of increased melanin production, which subsequently manifest as pigmented spots. Additionally, the impaired function of MC1R in individuals carrying *SPATA33* rs35063026 results in skin with lower UV-shielding capacity, contributing to an increased risk of wrinkles due to collagen degradation mediated by UV-induced MMP.

Collectively, these findings highlight the complex interplay of genetic architecture, allelic heterogeneity, and environmental context in shaping skin ageing phenotypes, underscoring the necessity for population-aware analyses and functional studies to elucidate causal mechanisms.

## Ethnic and sex differences in genetic associations

Significant variation in genetic associations was observed across ethnic groups. For instance, IRF4 rs12203592 was associated with skin ageing phenotypes such as pigmented spots and wrinkles in the Dutch and Latin American populations, but was absent in East Asian cohorts. This observation is likely due to the East Asian population, such as Chinese and Japanese, being homozygous for the C alleles of *IRF4* rs12203592, while the European population commonly carry the T allele (Sturm and Duffy 2012). Hence, carriers of the T allele are more vulnerable to the damaging effects of UV radiation due to reduced tanning capacity (Zhang et al. 2013). which may increase the risk of pigmented spots due to heightened UV sensitivity.

Although the *OCA2* gene is implicated in pigmentation across diverse populations, the specific variants associated with hyperpigmentation differ by ancestry. In the East Asian population, particularly among Korean and Chinese cohorts, missense variants of OCA2, which are the T allele of rs74653330 and rs1800414, are consistently associated with a protective effect against solar lentigines, while these variants are virtually absent in populations outside East Asian. This could be due to the fact that the C allele of rs1800414 is found almost exclusively in East Asia, with a frequency ranging from 62 to 76.1%. Outside of East Asia, this allele occurs at very low frequencies in some European populations, ranging from 1 to 3.6% Therefore, this may explain why the recessive allele of rs1800414 (i.e., the T allele) contributes a protective effect against pigmented spots in the Han Chinese population.

In contrast, other *OCA2* variants, such as rs17650960 and rs1498519, were reported in studies conducted in European cohorts to be associated with pigmented spots. However, these studies did not specify whether the observed associations corresponded to a risk-increasing or risk-reducing effect, highlighting the need for further studies to clarify the directionality of these associations in the European population.

Additionally, the overrepresentation of female participants (72.65%) across most studies may limit the generalisability of results. Moreover, none of the included studies stratified their findings by sex, highlighting the need for future sex-stratified GWAS to elucidate gender-specific ageing mechanisms.

## Limitations

While this systematic review synthesises valuable insights into genetic associations with skin ageing phenotypes, several important limitations warrant consideration. A key challenge lies in the substantial methodological heterogeneity across studies, including variability in phenotype definitions (e.g., pigmented spots versus solar lentigines), diverse statistical approaches (employing odds ratios, beta values, or effect sizes), and inconsistent significance thresholds. This variability complicates direct comparison of results and may affect the robustness of our conclusions. Notably, many studies failed to explicitly specify reference or alternative alleles, creating potential interpretation challenges, as exemplified by conflicting reports regarding *SPATA33* rs35063026 and *IRF4* rs12203592, where apparent discrepancies might simply reflect differences in allele reporting conventions.

Perhaps more significantly, the current literature provides limited biological interpretation for most identified genetic associations. While we have catalogued these variants in the Supplementary Table 1, their mechanistic roles in skin ageing processes remain largely unexplored. This represents a critical knowledge gap, as understanding the functional pathways linking genetic variants to phenotypic outcomes is essential for translating associations into meaningful biological insights. Future research should prioritise: (1) standardised reporting practices for genetic analyses, (2) functional validation of identified variants, and (3) investigation of population-specific effects that may modulate these relationships. Only through such targeted investigations can we move beyond correlation to establish causation in skin ageing genetics.

## Conclusion

This systematic review elucidates the multifaceted nature of skin ageing, revealing how intricate gene-environment interactions shape phenotypic outcomes through key biological pathways, including collagen metabolism, melanogenesis, oxidative stress response, and dermal structural integrity. Our synthesis highlights several critical insights: First, the recurrent association of *OCA2* missense variants (rs1800414, rs74653330) with reduced hyperpigmentation suggests a protective mechanism mediated through melanosome pH dysregulation, offering potential therapeutic targets for pigmentary disorders. Second, the pleiotropic effects of *SPATA33* (rs35063026) and *IRF4* (rs12203592) exemplify how single genetic variants can simultaneously influence wrinkle formation and pigmentation, providing a unified framework for understanding skin ageing's interconnected phenotypes.

Notably, we identified paradoxical variant-gene relationships that challenge conventional functional assumptions. Polymorphisms in hydration-related genes (*SPTLC1* rs7042102 and *REEP3* rs11961184) unexpectedly associated with increased wrinkle risk may reflect undiscovered roles in extracellular matrix maintenance. Similarly, the complex, context-dependent effects of *TERT* variants (rs2736098, rs2853669) on non-facial solar lentigines underscore the need to explore telomerase's non-canonical functions in pigmentation. The contrasting associations of AHR-mediated wrinkle formation versus CYP1A1-linked skin sagging, despite their shared collagen degradation pathways, further emphasise the precision required in mechanistic investigations.

While this review advances biological interpretations for many associations, substantial knowledge gaps persist. Nearly 40% of identified variants lack functional characterisation, and inconsistent reporting of allele effects complicates cross-study validation. Future research should advance our understanding of skin ageing by leveraging 3D skin models to investigate genetic variants with multiple effects, examining gene-environment interactions across diverse populations, and integrating multi-omics approaches to reveal underlying biological networks. These efforts will bridge genetic discoveries to personalised anti-ageing interventions and targeted therapies.

## Supplementary Information

Below is the link to the electronic supplementary material.

Supplementary file1 (DOCX 52 KB)

## Data Availability

No datasets were generated or analysed during the current study.
