# Supplementary material for: Deciphering the molecular clock: exploring molecular mechanisms and genetic influences on skin ageing
Source: Biogerontology. 2025 Aug 2;26(4):153. doi: 10.1007/s10522-025-10296-x (PMC12317886; doi:10.1007/s10522-025-10296-x)
Supplement: Supplementary file 1 — Supplementary file1 (DOCX 52 KB) [file 10522_2025_10296_MOESM1_ESM.docx]

**Supplementary Table 1:** Genes and SNPs without biological Interpretation for the association with the Skin Ageing Phenotype

| **Gene** | **SNPs** | **Skin Ageing Phenotypes** | **Function** |
| --- | --- | --- | --- |
| *ABMP* | rs7034903, rs12377342 | Facial solar lentigines | - Promote wound healing by promoting angiogenesis, collagen deposition and remodelling (Idrovo et al. 2015). |
| *ARHGEF7* | rs3825460 | Pigmented spots on the cheek | - Vital for axon formation and glomerular function (López Tobón et al. 2018; Matsuda et al. 2020). |
| *CAB39L* | rs2407616 | Sagging | - Function as a novel tumour suppressor in gastric cancer (Li et al. 2018). |
| *CBWD1* | rs478882 | Wrinkles | - Associated with melanoma susceptibility (Zhang et al. 2018). |
| *CDC42BPA* | rs4653497 | Pigmentation | - *CDC42BPA* encodes serine/threonine protein kinase MRCK, a downstream effector of CDC42, a protein involved in cell cycle regulation (Perumal et al. 2016). |
| *CENPBD1* | rs139810560 | Pigmented spots | - No known function for CENBPD1 - CENP-B stabilizes centromeric chromatin and ensures proper chromosome segregation (Fachinetti et al. 2015). |
| *CNTLN* | rs12350739 | Pigmented spots | - Maintain centrosome cohesion during interphase in mitosis (Jing et al. 2016). |
| *CTB-12O2.1, LINC01470* | rs10476781 | Wrinkles | - No known function |
| *DEF8* | rs139810560 | Pigmented spots | - Regulates cellular homeostasis in the nervous system (Oyarce-Pezoa et al., 2023) and is associated with actinic keratosis (Kim et al. 2022). |
| *DLGAP1* | rs4076011  rs8096287  rs11876749 | Eyelid sagging | - Related to brain disorders such as schizophrenia (Rasmussen et al. 2017). |
| *DOCK8, DOCK8- AS1* | rs478882 | Wrinkles | - *DOCK8* is critical for dendritic cell migration and plays a significant role in the survival and function of B cells, T cells and NK cells (Biggs et al. 2017). - No known function for DOCK8-AS1 |
| *FABP7* | rs6904500 | Facial solar lentigines | - Implicated in melanoma cell proliferation (Slipicevic et al. 2008). |
| *FCRL5* | rs117381658 | Wrinkle | - Involved in B-cell immune regulation (Damdinsuren et al. 2016). |
| *H2AFY2* | rs16927253  rs4746957  rs2271699  rs2394654  rs3750770 | Eyelid sagging | - Associated with chromatin remodelling and cellular stability (Mohammed Ismail et al. 2023). |
| *HLA-C* | rs2853949 | Facial solar lentigines | - Involved in immune recognition (Blais et al. 2011) and thought to regulate melanogenesis by eliminating aberrant melanocytes (Laville et al. 2019). |
| *HS3ST4* | rs59784607 | Pigmentation | - Involved in glycosaminoglycan biosynthesis (Lehri-Boufala et al. 2015). |
| *HSPA12A* | rs12259842 | Facial solar lentigines | - Member of the highly conserved 70-kDa heat shock protein (HSP70) family, which plays a crucial role in proteostasis by facilitating protein folding, multi-protein complex assembly, and transport (Radons 2016). |
| *RPS27P18, MRPS21P6* | rs4962295 | Wrinkle | - No known function |
| *MYH11* | rs76053540 | Nasolabial fold | - Components of the contractile apparatus in smooth muscle (Ruan et al. 2021). |
| *NCLN* | rs34466224 | Pigmentation | - Function as an antagonist of Nodal Signalling (Haffner et al. 2004). |
| *NMUR2* | rs10476781 | Pigmentation | - Promote cancer progression by enhancing the motility and invasiveness of cancer cells (Garczyk et al. 2017). |
| *SCEL* | rs912292 | Facial solar lentigines | - Involved in cornified envelope formation of keratinocytes (Baden et al. 2005). |
| *TERT* | rs2736098  rs2853672 | Non-facial solar lentigines | - Maintain telomere length to ensure chromosomal stability (Dratwa et al. 2020). |
| *TNFRSF6B* | rs1291206 | Wrinkle risk | - Promotes tumour progression by inhibiting apoptosis and immune surveillance (Zhang et al. 2021). |
| *TNFRSF8* | rs6690493 | Wrinkle risk | - Involved in lymphocyte activation and survival, immune response modulation, and tumour progression (Dumitru et al. 2023). |
| *TNS1* | rs11685354 | Pigmentation | - Regulates key cellular processes such as adhesion, migration, polarization, proliferation, and apoptosis (Wang et al. 2022). |
| *UNCX* | rs76548385 | Pigmentation | - Promotes the proliferation of basal progenitor cells in the olfactory sensory neuron (Sammeta et al. 2010). |
| *ZNF276* | rs35096708 | Pigmented spots | - Encodes zinc finger protein 276 which is highly expressed in breast cancer tissues and cell lines (Lei et al. 2022). - Associated with red hair trait (Farré et al. 2023). |

**References for supplementary data:**

Baden HP, Champliaud M-F, Sundberg JP, Viel A (2005) Targeted deletion of the sciellin gene resulted in normal development and maturation. genesis 42:219–228. <https://doi.org/10.1002/gene.20133>

Biggs CM, Keles S, Chatila TA (2017) DOCK8 Deficiency: Insights into Pathophysiology, Clinical Features and Management. Clin Immunol Orlando Fla 181:75–82. <https://doi.org/10.1016/j.clim.2017.06.003>

Blais M-E, Dong T, Rowland-Jones S (2011) HLA-C as a mediator of natural killer and T-cell activation: spectator or key player? Immunology 133:1–7. <https://doi.org/10.1111/j.1365-2567.2011.03422.x>

Damdinsuren B, Dement-Brown J, Li H, Tolnay M (2016) B cell receptor induced Fc receptor-like 5 expression is mediated by multiple signaling pathways converging on NF-κB and NFAT. Mol Immunol 73:112–121. <https://doi.org/10.1016/j.molimm.2016.04.001>

Dratwa M, Wysoczańska B, Łacina P, et al (2020) TERT—Regulation and Roles in Cancer Formation. Front Immunol 11:589929. <https://doi.org/10.3389/fimmu.2020.589929>

Dumitru AV, Țăpoi DA, Halcu G, et al (2023) The Polyvalent Role of CD30 for Cancer Diagnosis and Treatment. Cells 12:1783. <https://doi.org/10.3390/cells12131783>

Fachinetti D, Han JS, McMahon MA, et al (2015) DNA sequence-specific binding of CENP-B enhances the fidelity of human centromere function. Dev Cell 33:314–327. <https://doi.org/10.1016/j.devcel.2015.03.020>

Farré X, Blay N, Cortés B, et al (2023) Skin Phototype and Disease: A Comprehensive Genetic Approach to Pigmentary Traits Pleiotropy Using PRS in the GCAT Cohort. Genes 14:149. <https://doi.org/10.3390/genes14010149>

Garczyk S, Klotz N, Szczepanski S, et al (2017) Oncogenic features of neuromedin U in breast cancer are associated with NMUR2 expression involving crosstalk with members of the WNT signaling pathway. Oncotarget 8:36246–36265. <https://doi.org/10.18632/oncotarget.16121>

Haffner C, Frauli M, Topp S, et al (2004) Nicalin and its binding partner Nomo are novel Nodal signaling antagonists. EMBO J 23:3041–3050. <https://doi.org/10.1038/sj.emboj.7600307>

Idrovo J-P, Yang W-L, Jacob A, et al (2015) Combination of Adrenomedullin with Its Binding Protein Accelerates Cutaneous Wound Healing. PLOS ONE 10:e0120225. <https://doi.org/10.1371/journal.pone.0120225>

Jing Z, Yin H, Wang P, et al (2016) Centlein, a novel microtubule-associated protein stabilizing microtubules and involved in neurite formation. Biochem Biophys Res Commun 472:360–365. <https://doi.org/10.1016/j.bbrc.2016.02.079>

Kim Y, Yin J, Huang H, et al (2022) Genome-wide association study of actinic keratosis identifies new susceptibility loci implicated in pigmentation and immune regulation pathways. Commun Biol 5:1–9. <https://doi.org/10.1038/s42003-022-03301-3>

Laville V, Le Clerc S, Ezzedine K, et al (2019) A genome wide association study identifies new genes potentially associated with eyelid sagging. Exp Dermatol 28:892–898. <https://doi.org/10.1111/exd.13559>

Lehri-Boufala S, Ouidja M-O, Barbier-Chassefière V, et al (2015) New Roles of Glycosaminoglycans in α-Synuclein Aggregation in a Cellular Model of Parkinson Disease. PLoS ONE 10:e0116641. <https://doi.org/10.1371/journal.pone.0116641>

Lei T, Zhang W, He Y, et al (2022) ZNF276 promotes the malignant phenotype of breast carcinoma by activating the CYP1B1-mediated Wnt/β-catenin pathway. Cell Death Dis 13:781. <https://doi.org/10.1038/s41419-022-05223-8>

Li W, Wong CC, Zhang X, et al (2018) CAB39L elicited an anti-Warburg effect via a LKB1-AMPK-PGC1α axis to inhibit gastric tumorigenesis. Oncogene 37:6383–6398. <https://doi.org/10.1038/s41388-018-0402-1>

López Tobón A, Suresh M, Jin J, et al (2018) The guanine nucleotide exchange factor Arhgef7/βPix promotes axon formation upstream of TC10. Sci Rep 8:8811. <https://doi.org/10.1038/s41598-018-27081-1>

Matsuda J, Maier M, Aoudjit L, et al (2020) ARHGEF7 (β-PIX) Is Required for the Maintenance of Podocyte Architecture and Glomerular Function. J Am Soc Nephrol JASN 31:996–1008. <https://doi.org/10.1681/ASN.2019090982>

Mohammed Ismail W, Mazzone A, Ghiraldini FG, et al (2023) MacroH2A histone variants modulate enhancer activity to repress oncogenic programs and cellular reprogramming. Commun Biol 6:1–19. <https://doi.org/10.1038/s42003-023-04571-1>

Oyarce-Pezoa S, Rucatti GG, Muñoz-Carvajal F, et al (2023) The autophagy protein Def8 is altered in Alzheimer’s disease and Aβ42-expressing Drosophila brains. Sci Rep 13:17137. <https://doi.org/10.1038/s41598-023-44203-6>

Perumal D, Lagana’ A, Melnekoff D, et al (2016) Network Modeling Reveals *CDC42BPA* and *CLEC11A* As Novel Driver Genes of t(4; 14) Multiple Myeloma. Blood 128:802. <https://doi.org/10.1182/blood.V128.22.802.802>

Radons J (2016) The human HSP70 family of chaperones: where do we stand? Cell Stress Chaperones 21:379–404. <https://doi.org/10.1007/s12192-016-0676-6>

Rasmussen A, Rasmussen H, Silahtaroglu A (2017) The DLGAP family: Neuronal expression, function and role in brain disorders. Mol Brain 10:. <https://doi.org/10.1186/s13041-017-0324-9>

Ruan J, Zhang L, Hu D, et al (2021) Novel Myh11 Dual Reporter Mouse Model Provides Definitive Labeling and Identification of Smooth Muscle Cells—Brief Report. Arterioscler Thromb Vasc Biol 41:815–821. <https://doi.org/10.1161/ATVBAHA.120.315107>

Sammeta N, Hardin DL, McClintock TS (2010) Uncx regulates proliferation of neural progenitor cells and neuronal survival in the olfactory epithelium. Mol Cell Neurosci 45:398–407. <https://doi.org/10.1016/j.mcn.2010.07.013>

Slipicevic A, Jørgensen K, Skrede M, et al (2008) The fatty acid binding protein 7 (FABP7) is involved in proliferation and invasion of melanoma cells. BMC Cancer 8:276. <https://doi.org/10.1186/1471-2407-8-276>

Wang Z, Ye J, Dong F, et al (2022) TNS1: Emerging Insights into Its Domain Function, Biological Roles, and Tumors. Biology 11:1571. <https://doi.org/10.3390/biology11111571>

Zhang C, Li H, Huang Y, et al (2021) Integrative analysis of TNFRSF6B as a potential therapeutic target for pancreatic cancer. J Gastrointest Oncol 12:. <https://doi.org/10.21037/jgo-21-303>

Zhang T, Choi J, Kovacs MA, et al (2018) Cell-type–specific eQTL of primary melanocytes facilitates identification of melanoma susceptibility genes. Genome Res 28:1621–1635. <https://doi.org/10.1101/gr.233304.117>
